# One Health as a potential platform to rescue the neglected fruit trees in Yucatan, Mexico

**DOI:** 10.1016/j.soh.2024.100073

**Published:** 2024-07-23

**Authors:** John P. Ehrenberg, Afona Chernet, Manuel Luján, Jürg Utzinger

**Affiliations:** a Avenida Cedro 9, # 303, Cholul, Merida, Yucatan, 97305, Mexico; b Retired, World Health Organization, Manila, 1000, Philippines; c Swiss Tropical and Public Health Institute, CH-4123 Allschwil, Switzerland; d University of Basel, CH-4001 Basel, Switzerland; e Royal Botanic Gardens, Kew, Richmond, Surrey, TW9 3AB, UK

**Keywords:** Ethnic minority groups, Mayan fruit trees, Mexico, Nature-based solutions, Neglected and underutilized species of plants, Neglected tropical diseases, One Health, Sustainable Development Goals

## Abstract

Neglected and underutilized species of plants (NUS) have been identified by the Food and Agriculture Organization as valuable resources for fighting poverty, hunger and malnutrition as they can help make agricultural production systems more sustainable and resilient. Adaptation of NUS to changing environments over several millennia has rendered most of these plants resistant to pests and climate change. In this paper, we explore the potential values of some of the Mayan fruit trees justifying conservation efforts in their native habitats. Our research was primarily based on a scoping review using Google Scholar. We considered articles published in English, Spanish and Portuguese. Our review rendered two sets of articles including those focusing on the nutritional and medicinal properties of NUS and their products, and those focusing on their uses in traditional medicine. Both sets of papers strongly support arguments for conservation of NUS. Additionally, our scoping review expands and includes a case study on the conservation of NUS, highlighting the critical role of civil society on how it can spearhead rescue efforts of botanical resources through the creation of what is possibly the first arboretum of its kind in the Americas. Among the project's key selling points was not only the rescue of an important component of Yucatan's cultural heritage but its nutritional value as well as its potential medicinal properties. Our paper is not prescriptive on how to preserve or even commercially exploit NUS. It is intended as a thought-provoking piece on the potential of a One Health approach as a multisectoral platform to support conservation efforts, while stimulating greater interest in the subject and encouraging more action from the academic and pharmaceutical sectors as well as civil society.

## 1 Background

Impressive scientific achievements have been made over the past decades in most disciplines. Yet, there are many challenges and obstacles to achieving the Sustainable Development Goals (SDGs) [1]. According to a new study put forth by the National Aeronautics and Space Administration, production of the world's major staple crops will likely decline over the next few decades [2], especially maize, an important staple crop in many low- and middle-income countries (LMICs). Global food security is also threatened by genetic erosion, a term coined by the Food and Agriculture Organization (FAO) to describe the loss of genetic resources due to the preferential large-scale cultivation of a few high-yielding cultivars in detriment of their wild relatives [3].

While large-scale agricultural development systems have contributed to a significant increase in the availability of critical crops to feed an ever-increasing human and animal population, this has come at a cost, including deforestation, depletion of freshwater reserves and severe environmental pollution with pesticides, fungicides and fertilizers to the detriment of human and animal health. Furthermore, some of these systems lack proper environmental, social and health impact assessments and are severely transforming tropical forests resulting in massive biodiversity loss worldwide [4]. Genetically modified (GM) crops have been the focus of intense scrutiny and controversies on their potential negative impact on human and animal health (e.g. toxicity, allergenicity and genetic hazards) [5]. Importantly, upon implementing conservation efforts, more attention needs to be paid to crops and useful wild species that are locally or regionally more appropriate for human and animal consumption [6]. These species have been described as orphan crops or neglected and underutilized species of plants (NUS). Although NUS are not traded as commodities, they have been identified by FAO as valuable resources for fighting poverty, hunger and malnutrition as they can help make agricultural production systems more sustainable and resilient. An example of such resilient NUS is *Brosimum alicastrum*, the foliage of which is widely used in animal feed in many parts of Mexico, especially in the Yucatan Peninsula during severe drought conditions [7]. Furthermore, the fruits were and continue to be much appreciated for human consumption, particularly by indigenous Mesoamerican people [8]. Indeed, NUS have shown significant nutritional benefits with considerable potential to alleviate global micronutrient deficiencies [9]. Furthermore, many NUS are key ingredients in traditional foods that play a critical role in maintaining the cultural identity of indigenous communities [10]. This is one of the key arguments behind its conservation.

Addressing the conservation of NUS and their products requires an intersectoral approach to tackle the multiple issues faced by decades of neglect following the same rationale as the prevention, control and elimination of neglected tropical diseases (NTDs) [11]. At stake are the ecosystem's services they render as a whole and the health of the planet.

The purpose of this piece is to draw attention of multiple stakeholders across different sectors towards the gradual disappearance of a botanical resource, Mayan fruit trees. Though not exhaustive, our paper lists some of the nutritional and medicinal properties of NUS as well as some of its uses in traditional medicine, potentially leading to the development of new drugs to treat human and animal diseases. Our paper also summarizes the experiences of a small-scale rescue project conducted in Yucatan, Mexico [12] – Mayan Fruit Trees Arboretum – focused on the rescue of Mayan fruit trees and on the restoration of traditional Mayan village backyards through community involvement. The role and responsibilities of different stakeholders, especially civil society, in rescuing NUS is highlighted throughout the paper, strengthening the argument for their potential inclusion into One Health.

## 2 Methods

### 2.1 Scoping review

Information for this article was primarily based on a desktop-based scoping review of relevant articles on 25 different autochthonous fruit trees belonging to 21 different genera published in English, Spanish and Portuguese. We used Google Scholar with the following search terms: medicinal uses, traditional uses, indigenous fruits, specific names of fruit trees, home gardens, guide to edible wild fruits, Mayan fruits and native fruit tree arboreta. The review was compiled over a period of five weeks (May 22 through June 23, 2023). The authors also tapped on the information from a book recently published by the first and corresponding author of the current article, which relied on an extensive search using EBSCO platform, and the University of Yucatan's own bibliographic collection [12].

### 2.2 Case study: the Mayan Fruit Tree Arboretum

The rescue of Mayan fruit trees began approximately 35 years ago with the collection of different fruits and fruit tree species encountered during several field trips across the State of Yucatan. Although the range of these trees extends well beyond the Maya region, these were some of the fruits that the Mayans used to consume and some still do. These trees later provided most of the seedlings for the establishment of the Mayan Fruit Tree Arboretum in the community of Cholul at the outskirts of the capital city of Merida in Yucatan.

The collective experience of a group of volunteers in galvanizing the involvement of both the community as well as the public sector has been one of leading by example. Several community members were invited to participate in various training sessions offered by the co-founders of the Mayan fruit tree rescue project, from preparing compost, collecting and germinating seeds to establishing small tree nurseries in people's home gardens.

In February 2018, this group launched the project with the involvement of 72 volunteers from the community to cultivate some of the neglected native fruits in an area expanding over 700 m^2^ within the boundaries of a public park in the community of Cholul, on the outskirts of the city of Merida, State Capital of Yucatan [12]. The group planted 25 different native fruit tree seedlings belonging to 21 different genera and 25 species.

### 2.3 Rescuing NUS: potential of a One Health platform

One Health could offer a platform for the purpose of rescuing NUS as an innovative initiative, as it bridges the communication and collaboration divide between human and animal health and the environmental sectors. It could also promote synergies with other equally neglected issues, some of which have already attracted attention with palpable results. Such is the case of One Health surveillance and response for early detection of emerging, re-emerging or exotic zoonotic pathogens [13]. One Health is actually already laying the ground for the integration of plant health and its relevance to food production by harnessing its collaborative and holistic potential. Discussions are under way for its possible role in the transformation and improvement of food systems in the Americas [14]. The recent One Health in the Americas Conference focused on foodscapes and health and highlighted, for example, the value of local cultures as is the case of good food markets, a mission-driven grocery store that aims to provide fresh food to people living in communities with very limited access to fresh produce (i.e. “food deserts”) [14]. The case study presented here is already relying on the health impacts of such a project to sensitize the community and the public sector towards the importance of preserving these fruit trees.

## 3 Results

### 3.1 Key findings from the scoping review

The key findings of our scoping review are summarized in Table 1. The review rendered two large sets of articles. A first set of articles focused on the nutritional and medicinal properties of NUS and related products. The second tier of articles discussed the uses of NUS in traditional medicine. It is this collection of fruit trees that was used to establish the Mayan Fruit Tree Arboretum in the community of Cholul.

**Table 1:** Some of the nutritional and medicinal properties of Mayan fruits, tree bark, leaves and seeds and their uses in traditional medicine.^1^

Tree	Nutritional and medicinal properties	Use in traditional medicine
*Pouteria campechiana* (Kunth) Baehni [15]	1. Rich in essential mineral contents, copper, zinc, iron, potassium and vitamin C 2. Rich on fiber, protein, phenolic, flavonoids with potential anti-oxidant, anti-inflammatory and cytotoxic effects 3. *P. campechiana* seeds have been found to have potent anti-microbial activity against both Gram-positive and Gram-negative bacteria	1. The decoction from *P. campechiana* bark is utilized as an antipyretic in Mexico and to treat skin blisters and soreness in Cuba 2. Unripe fruit is ingested for diarrhoea 3. The leaves are reported to be anti-inflammatory 4. *P. campechiana* seeds are also used as a remedy for ulcers
*Psidium guajava* L. [16,17]	1. Multiple phytochemicals have been isolated from guava leaves with potential anti-cancer, anti-diabetic, anti-oxidant, anti-diarrhoeal and anti-microbial properties 2. Rich presence of minerals and proteins, as well as vitamins 3. Guava leaves also contain many secondary metabolites, such as flavonoids, triterpenoids, sesquiterpenes, glycosides, alkaloids, saponins and other phenolic compounds, with a potential activity as immune stimulators and modulators of chronic diseases	1. Wide range of uses depending of the region of the world: leaves, seeds and fruit might be used to treat any condition including respiratory and digestive problems, as an anti-spasmodic, anti-inflammatory, control of hypertension, diabetes to sores, cuts, sprains, boils, rheumatism, seizures, among other uses
*Chrysophyllum cainito* L. [18,19,20]	1. The fruit exhibits anti-oxidant properties due to the presence of polyphenols. Other phyto-constituents present include alkaloids, glycosides, triterpenoids and sterols 2. Pectin is present in the extracts and fractions of the pulp and is widely used in many food and pharmaceutical industries as a gelling agent 3. Studies have reported the presence of different pharmacological substances such as anti-oxidant, anti-diabetic, anti-microbial and anti-inflammatory. Their mechanism of action is unknown	1. Extracts from the leaves, stem bark, fruits, peel, pulp or seed of *C. cainito* are used in traditional medicine for curing diabetes, as well as bacterial, fungal and viral infections 2. Mature pulp is used to treat soar throat and when the fruit is still green it is used to treat constipation
*Pouteria sapota* (Jacq.) H.E. Moore & Stearn [21,[22], [23], [24]]	1. Mamey contains a variety of carotenoids including novel ketocarotenoids, an important pro-vitamin A source 2. It contains coumarins with insecticidal properties 3. Rich on carbohydrates providing 134 calories per 100 g and fibers to improve intestinal transit 4. Contains vitamin C with anti-oxidant properties 5. Contains extracts with anti-bacterial properties	1. It is widely used in traditional medicine throughout Mexico, from treating hair loss to preventing dandruff 2. It is used to treat digestive disorders, including diarrhoea and stomachache 3. The roasted seeds are applied to wounds 4. The powder of roasted seeds is diluted in water and drunk to treat asthma and cough 5. It is used to treat heart conditions and scabies
*Couepia polyandra* (Kunth) Rose [25,[26], [27], [28]]	1. Members of the Chrysobalanaceae family are rich on triterpenoids, diterpenoids, steroids and phenylpropanoids like flavonoids 2. Olosapo is an excellent source of fiber, which can help stimulate the digestive tract 3. The fruits also contain vitamin C, an anti-oxidant that can help strengthen the immune system, boost collagen production within the skin and reduce inflammation	1. Leaves and bark are used to clean eyes and wounds 2. In the State of Veracruz it is used to prevent pregnancy in combination with a number of herbs
*Annona purpurea* Moc. & Sessé ex Dunal [29]	1. Annona is the most studied genus out of a total of 120 genera of the Annonaceae family and contains more than 119 species of trees and shrubs, 105 of which are found in tropical America 2. Fruits are edible and most are extremely tasty yet only a limited number of these species have economic value, including *A. squamosa*, *A. muricata*, *A. reticulata*, *A. glabra*, and *A. purpurea* 3. Fruits, seeds, leaves and bark have been found to contain several classes of secondary metabolites, including acetogenins, essential oils, alkaloids, terpenoids and flavonoids with anti-bacterial, anti-cancer, anti-diabetic and anti-inflammatory properties	1. Depending on the region of the world, infusions, pastes and decoctions might be used for wound healing, as analgesics, to treat dysentery and as an anthelminthic medicine 2. Leaves and bark might be used to treat fever, asthma and cough 3. Bark and roots might also be used as anti-spasmodic and to lower blood pressure
*Annona reticulata* L. [30,31]	1. Fruits, leaves and bark contain tannins, alkaloids, phenols, glycosides, flavonoids and steroids 2. Medicinal properties include its action as anthelminthic, analgesic, anti-inflammatory, antipyretic and wound healing agent	1. *A.reticulata* is used in ayurvedic medicine to treat epilepsy, dysentery, cardiac problems, worm infestation, constipation, haemorrhage and various bacterial infections, to mention a few 2. Mayans use fresh leaves to treat joint pain (rheumatism) 3. They also use the leaves to treat human and chicken ectoparasites (lice)
*Annona squamosa* L. [32,33]	1. Rich in bioactive compounds 2. Seed extract exhibit activity against *B. subtilis*. 3. Peel extracts exhibit anti-microbial (e.g. against *K. pneumoniae*) and anti-fungal activity 4. Leaf extract exhibit activity against *Salmonella typhi*, *S. aureus*, *Pseudomonas aeruginosa* and *B. subtilis* 5. High on vitamin C (35–42 mg/100 g), higher than that of grapefruit 6. Fruit is also high on thiamine and potassium 7. Seeds contain annonastatin 8. Bark contains acetogenin	1. Leaves are used in traditional medicine as a vermicide 2. They are also applied to abscesses, insect bites and other skin conditions including ulcers and wounds 3. These might also be used as pain relievers including joint pain 4. Scrapings of root-bark are used for toothache 5. Powdered seeds are used to kill head-lice and fleas 6. Fruits are used to treat stomachache and colds
*Annona muricata* L. [34,35]	1. Sour sop fruit consists of 67.5% edible pulp, which contains 80%–81% water, 1% protein, 18% carbohydrate, 3.4% treatable acidity, 24.5% non-reducing sugar, and vitamins B1 B2 and C 2. Secondary metabolites have been isolated and identified, including acetogenins, alkaloids, phenolic compounds and megastigmanes with anti-inflammatory, anti-cancer and other immune system related effects 3. The sour sop fruit's major use throughout the tropics is as a juice where it is widely used and processed in various presentations (even canned)	1. *A. muricata* is widely used in traditional Indian medicine for the "treatment of kidney troubles, fever, nervousness, ulcers and wounds" and possesses anti-spasmodic, anti-dysenteric and parasiticidal activity 2. Its bark and roots are used to treat stomach problems in some rural communities of the Yucatan Peninsula 3. The leaves and bark are also used to treat asthma and coughs 4. In some rural communities of the Yucatan Peninsula, branches are used to treat “evil eye” and the leaves to wash the hair, and treat nasal bleeding
*Cordia dodecandra* A. DC. [[36], [37], [38], [39]]	1. Results of a study conducted in Yucatan, Mexico showed that a dichloromethanic extract of *C. dodecandra* bark induces relaxation of smooth muscle mainly by cAMP increase and calcium channel blockade, and hence, might be potentially useful in the treatment of chronic respiratory diseases such as asthma 2. Phenolic, alkaloids, and terpenoids scavenge free radicals, which have been related to the development and complication of diabetes mellitus	1. Leaves, fruit, bark and seed of most of *Cordia* species are widely used in ethno-botanical medicine 2. Leaves are used most frequently to treat many ailments from respiratory disorders to diarrhoea
*Bixa orellana* L. [[40], [41], [42]]	1. A long list of biological activities attributable to the seeds has been tested in several countries and models 2. Leaf extracts have been found to have a broad action against multiple bacteria and fungi, including *Salmonella typhi*, *Acinetobacter*, *Bacillus subtilis*, *Staphylococcus aureus*, *Streptococcus pyogenes*, *Pseudomonas aeruginosa*, *Escherichia coli* and *Candida albicans* 3. The therapeutic properties of the seeds (e.g. anti-oxidant and hypoglycemic) have been attributed to its high levels of carotenoids, including bixin, a red-colored carotenoid while the main oil constituent is geranylgeraniol 4. Seeds are ground and used extensively in Mayan dishes as well as famous Indian dishes such as Tandoori chicken	1. Known as “urucum” by the Tupi people of Amazonia, it is used, depending of the country of the world, as anti-pyretic, cardiotonic, anti-diarrhoeal, as insect repellent, laxative, to treat burns, as aphrodisiac, to treat gonorrhea and dysentery
*Diospyros nigra* (J.F.Gmel.) Perr [[43], [44], [45]]	1. It contains phenolic compounds and carotenoids which give it its characteristic dark brown to black colour 2. It also contains tannins and various nutrients including vitamin C, vitamin E and calcium 3. Its compounds have anti-oxidant properties at different stages of fruit growth 4. Black zapote, as is the case of the persimmon, produces high amounts of carbon dioxide (CO_2_) and ethylene (C_2_H_4_), which makes this fruit highly perishable, even more so than the persimmon, hence its rarity in supermarkets even in their own home ranges 5. A related African species *D. lycioides* Desf. Extracts demonstrated among others, anti-inflammatory, anti-oxidant, anti-fungal and anti-bacterial activities	1. It is widely used throughout most of the countries where the species is native such as in the Americas and introduced including tropical Africa 2. It is used throughout this range for various purposes, including treatment of abdominal pains, infertility in women, sexually transmitted infections, and anaemia and as chewing sticks or toothbrush 3. The bark is soaked, ground and boiled to treat diarrhoea 4. The peel is soaked and consumed as a refreshing drink but it is also believed to be effective to treat leprosy and itching
*Coccoloba uvifera* (L.) L. [[46], [47], [48]]	1. Diverse phytochemicals contained in the fruit may have free radical scavenging and anti-oxidant characteristics, as is the case of other neglected Mayan fruits 2. Fruits are rich in copper, iron, potassium and manganese as well as a good source of vitamins A, B, C and K and beta-carotene 3. Search of natural anti-mutagens is opening up new and interesting venues in cancer treatment 4. This fruit and its by-products could represent a promising field in anti-mutagen research	1. The bark is used to treat diarrhoea 2. Leaves are thought of being diuretic 3. The fruit is placed over the eyes to sooth burning sensation and relax the lids 4. Decoction of leaves is used to treat asthma and hoarseness 5. It is also used to wash wounds and as an astringent
*Melicoccus bijugatus* Jacq [49,50]	1. In Latin America, the fruit is processed to make jams, jellies, preserves, juices, sauces and fillings. The seeds may also be roasted and eaten 2. The fruit is a source of fiber, vitamins A, B and C, calcium, iron and tryptophan 3. It is also a source of various phenolics, flavonoids and their associated sugar derivatives 4. Procyanidins are thought to contribute to the medicinal properties of the seed extracts 5. Phenolic compounds are associated with several health benefits which include the prevention of diabetes, cardiovascular disease as well as mitigating symptoms of gastrointestinal disorders 6. Coumaric acid is a systemic anti-oxidant with anti-platelet activity in humans 7. Pulp extracts exhibited anti-microbial activity	1. A blend of roasted and ground seeds is prepared in some countries of Latin America and mixed with boiled leaves and bark to treat fever and diarrhoea 2. The fruits are used to treat gastrointestinal and respiratory problems
*Melicoccus oliviformis* Kunth [51]	1. There are quite a few articles on its taxonomy and subspecies but very few if any on other topics 2. A comprehensive study was done as part of a thesis project to determine phito-components of several wild occurring fruit trees, *M. oliviformis* among these 3. All were found to have a high sugar content, which spider monkeys seem to prefer over other fruits with less carbohydrate contents 4. Gastrointestinal disorders such as indigestion, ulcers, diarrhoea, stomach pain and dysentery are among the most common ailments in rural areas in the States of Veracruz and Oaxaca 5. Causes are attributed to contaminated food, nutritional factors and pathogens (bacteria, viruses, parasites and helminths) 6. In a survey undertaken in Mexico (1983–1985) authors found that 50% of the locally available medicinal plants were used to treat these illnesses	1. In Mexico, infusions are prepared with their leaves to treat nervous disorders and fever associated with throat infections. It is also used to treat diarrhoea
*Byrsonima roigii* Urb. and *Byrsonima crassifolia* (L.) Kunth [52]	1. Fruits are a good source of vitamin C, total phenolic compounds and flavonoids with antioxidant capacity 2. High sugar content	1. The bark is used in traditional medicine as anti-parasitic, anti-bacterial, anti-fungal, anti-tumor, anti-inflammatory and anti-cough medicine, also as antipyretic 2. It is also used to treat asthma, bronchitis, some stomach ailments and even diabetes and malaria
*Manilkara zapota* (L.) P.Royen [53,54]	1. The fruit is rich in vitamins and polyphenolic anti-oxidants, which have a free radical scavenging activity 2. Natural anti-oxidants can assist in the protection of human cells against the effects of free radicals that may contribute to cancer forming cells among other diseases 3. Fruit also contains carbohydrate and minerals, a good source of energy 4. Leaves also contain flavonoid and bioactive polyphenolic compounds 5. Tannin from leaves extracts have been considered for use as an anti-microbial agent to extend the shelf life of food 6. They have also anti-hyperglycemic, hypocholesterolemic and anti-oxidant activity 7. Leaf ethanoic extracts possess anti-inflammatory activity	1. Applied against arthritic problems, for the relief of gastrointestinal problems and to relieve abdominal pain of menstrual origin 2. It was used as a diuretic in Mexico up to the 18th century
*Muntingia calabura* L. [[55], [56], [57]]	1. Flavonoids have been obtained from root extracts, chalcones and flavonoids from leaves 2. Some of these bioactive compounds have been found to have anti-oxidant, antipyretic, anti-platelet aggregation, cardio-protective, anti-bacterial and anti-inflammation properties 3. Leaves and stems contain phenolic compounds with anti-tumor properties	1. Depending on the geographical location, it is used for a large spectrum of ailments 2. As an anti-inflammatory and antipyretic agent 3. The roots are used as antiseptic and to treat stomach ailments 4. Flower infusions are used as tranquilizers, as tonic, to treat toothache headache and colds 5. The fruit is taken as a cough remedy and other respiratory ailments 6. In the Huasteca region of Mexico it is used to treat chickenpox and measles
*Spondias purpurea* L. [58,59].	1. Phytochemical screening in leave extracts revealed presence of flavonoids and epigallocatechin 2. Epigallocatechin has high anti-oxidant and anti-ulcerogenic capacities 3. Bark extracts were active against strains of *Staphylococcus aureus*, *Staphylococcus epidermidis*, *Pseudomonas aeruginosa*, *Bacillus cereus* and *Escherichia coli*	1. Leaves and bark extracts are used as antipyretic 2. Leaves infusion are used in Nigeria to treat wounds, skin burns and scab and flatulence in children 3. The sap is used in the Philippines to treat stomatitis in infants
*Parmentiera aculeata* (Kunth) Seem [60,61]	1. Anti-oxidant activity and anti-bacterial activity was obtained from ethanol extracts of ripe pulp as well as higher polyphenol content from metabolic extracts also from ripe pulp 2. A study evaluating these properties in rats reported the effectiveness of hexane and methanol extracts from the plant in promoting the fragmentation of bladder stones	1. Fruit, roots, bark and flower may be prepared as an infusion to be used for kidney pain and to treat urinary tract infections 2. All parts are used medicinally. In Mexico, a tea made with the root, bark and fruits is used to treat kidney ailments and urinary tract infections
*Pouteria glomerata* (Miq.) Radlk [62,63]	1. *P. glomerata* fruits have been found to have anti-oxidant capacity and high levels of vitamin C, minerals (mainly chromium, molybdenum and phosphorus), dietary fiber and malic acid 2. Consumption of dehydrated fruits of *P. glomerata* can provide a good amount of these nutrients, meeting daily needs 3. A total of 30 volatiles were identified in the fruit. Among these were ketones, terpenes, esters, alcohols, aldehydes and a sulfur compound 4. The interaction of these components contributes to the complexity of the flavour of the fruit resembling that of sweet potato 5. It has been suggested that due to its high content of scualene and secondary metabolic derivatives such as acetylated products of β-lupeol y β-amiryn, these fruits and their byproducts could eventually be used in the treatment of diabetes	1. It is used in some parts of Mexico to treat diabetes, nervousness, purify blood and whooping cough 2. In its wild state, it is known to be the principle host of many different types of fruit flies
*Pimenta dioica* (L.) Merr [64]	1. Contain many novel aromatic compounds, mostly glycosides and polyphenols with anti-bacterial, hypotensive, anti-neuralgic and analgesic properties 2. Two of its compounds, eugenol and gallic acid have selective anti-proliferative and anti-tumor properties on human cancer cells and animal models 3. New characterization of novel compounds such as ericifolin show potent anti-prostate cancer and anti-breast cancer properties	1. Medicinal preparations depend on the country 2. It is used for many purposes in Mexico among these, to reduce labor time, spasm, nausea, stomach ache, diarrhoea, as a tonic, stimulant, antiseptic and to relieve flatulence 3. Infusions of leaves are used to treat rheumatic pains, anti-diabetic or to lower the fever
*Mammea americana* L. [23,24,65]	1. Rich on coumarines, xanthones, triterpenes, tannins, alkaloids, flavonoids, and/or steroids, quinones and saponins 2. Anti-tumor activity 3. Anti-bacterial activity with *M. americana* extract on *P. gingivalis* and *S. mutans* (at lower minimum inhibitory concentration) 4. Depending on the concentration of the extract, bactericidal and bacteriostatic activity against *S. mutans* and bacteriostatic activity against *P. gingivalis*	1. Medicinal preparations depend on the country 2. Treatment of skin diseases, fever, inflammation and as an insect-repellent 3. Ethnomedical data showed that the bark is used to treat 14 conditions in Colombia, the most representative being gallstones, prostate inflammation and malaria

1 The reported nutritional and medicinal properties, and traditional medicine uses contained in this table are a sample of what has been reported in the literature and should not be considered as all inclusive.

These trees and their products, including fruits, leaves, bark and flowers are described in our extensive review as having a broad range of properties covering a wide spectrum of health conditions, such as anti-microbial agents, respiratory, digestive and metabolic diseases (Table 1). They have also been used for centuries and some still are by traditional healers in many indigenous communities throughout the world, especially in those lacking adequate health care facilities (see Table 1, section traditional medicine).

**Fig. 1:**
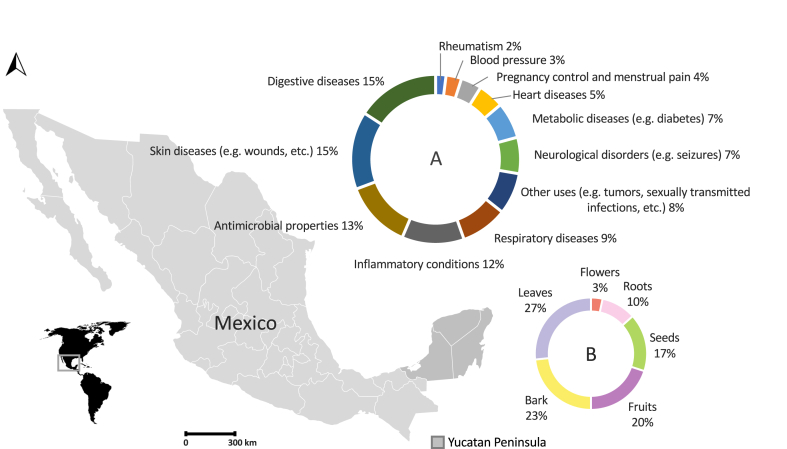
Traditional medicinal uses of native tree species in the Yucatan Peninsula of Mexico. A) Diseases and conditions most commonly treated. B) Plant parts most commonly used in traditional medicine.

Fig. 1A classifies the spectrum of traditional medicinal properties according to the percentage of consulted articles (Table 1), which report on the potentials of any given tree product to treat various health conditions. Information depicted in Fig. 1B was also obtained from our scoping review regarding the percentage of any of these tree parts which were used for traditional medicinal purposes. In this review, native tree plant parts were found to be used mainly to treat digestive (15%) and skin diseases (15%). Anti-microbial properties were reported in 13% of the reviewed articles, followed by a smaller percentage of articles reporting on the treatment of a broad spectrum of other diseases (Fig. 1A). Leaves were found to be the most commonly used plant part in traditional medicine (27%), followed by bark (23%), fruits (20%), seeds (17%), roots (10%) and flowers (3%) (Fig. 1B).

### 3.2 Case study

The arboretum project was established and has been sustained over a period of 6 years. All of the trees are thriving and some are already bearing fruits which any visitors can pick. The project is considered to be the first arboretum of its kind in a public space in Mexico and possibly in the Americas [12]. The arboretum has since attracted the attention of various sectors at the local, national and international level, and has been replicated in other parts of the city of Merida (e.g. other public parks and public schools) [12]. Moreover, the arboretum is bound to become a tourist attraction adding value to the other existing attractions in the park, including the adjacent XVI century colonial church and a small “cenote” (natural sink hole). The project offers the possibility of providing an income generating activity for local communities as well as the means to improve nutrition and health. It was documented in a digital book with free access, which is now circulating worldwide [12]. Few such initiatives are underway in Mexico, and elsewhere in the Americas. Hence, this may be one of the few relevant projects in the Americas that is focusing on the conservation of a group of NUS.

## 4 Discussion

Fruits are considered a tremendous potential resource for the development of innovative and healthy products for the food and pharmaceutical industries. Neotropical fruits have been used in traditional medicine for centuries mainly in their native habitats but increasingly so in other geographical areas beyond the Americas (Fig. 1 and Table 1). The wide range of uses in traditional medicine has paved the way for the discovery of active compounds of interest to the pharmaceutical and agricultural sectors (e.g. pesticides) (Table 1). Interestingly, some of its perceived benefits in traditional medicine seem to coincide with the properties of isolated phytochemicals, many of which have additional potential benefits such as myricitrin, one of the compounds isolated from the leaves of *Pouteria campechiana.* It possess a toxic effect against both *Aedes aegypti* and *Culex quinquefasciatus* mosquitoes at all stages [15]. Candles and soaps are prepared using the pulp of *Couepia polyandra* [12]. The natural dye of the seeds of *Bixa orellana* is widely employed worldwide. It is used in the textile, paint and cosmetic industries and it is also one among very few accepted by the World Health Organization (WHO) to be added to food products [66,40]. It has also been shown to have larvicidal properties against *Ae. aegypti* [67]. *Parmentiera aculeata* fruit holds promise as livestock feed [68]. All spice (*Pimenta dioica*) might also be used in the production of cosmetics and perfumes [69]. *Acrocomia aculeata* kernel can be used to obtain oil and as an ingredient in the manufacture of soap and cosmetics as well as biofuels [70]. It is obvious, there is ample room to further our knowledge and understanding regarding the multiple properties of these fruits and their products beyond their nutritional and medicinal values.

While North American Free Trade Agreement (NAFTA) was clearly beneficial on many fronts in Mexico, it did have a severe negative impact on the agricultural sector, affecting both products and farmers [71,72]. Unfortunately, as a result of the NAFTA and other free trade agreements, there has since been an increasing tendency to consume more of the exotic and less of the native products. We are facing a rather serious transculturation of people's tastes [73]. That is, a change in food consumption patterns at the expense of healthy food, including fruits [73]. Although this phenomenon needs to be properly documented, project leaders and principal author of the book noted that markets were offering less of the local fruit varieties, resulting from the villagers increasing reluctance to plant these over the course of barely three decades [12]. Home gardens function as *de facto* germplasm banks for most of these species of trees; hence, the need for sustaining traditional backyards [36].

### 4.1 Enriching Yucatan's biodiversity

Although various different plant species are consumed as grains, vegetables and fruits, only a few of these are commercialized. Numerous NUS offer the potential to complement the human diet as well as increasing the level of food production seeking more sustainable and resilient agricultural food systems as well as more economical ones [3,9,10]. Backyards (solares in the Latin American context) were common throughout many Latin American countries. The practice dates back to pre-Hispanic times. They provided, and in some instances still do, households with a good amount of fruits, vegetables, honey and domestic animals, including turkeys (domesticated in central Mexico) as well as medicinal plants that were and are often still used in traditional celebrations [74]. Urbanization over merely three decades has caused backyards to vanish, especially in peri-urban environments, but also increasingly in rural settings throughout Mexico and in many other countries of the Americas [75].

Some of the fruits trees (e.g. *Manilkara zapota*) still exist in their wild form throughout the American tropics, though less and less so. Of note, the very first chewing gum was produced by processing the zapote's sap. It is an interesting and relevant story and lesson on how a native fruit tree (now neglected) yielded one of the highest profitable tree products in the world at the time (i.e. around 1860). Not only the fruit, but indeed the whole history of its best known product obtained from its latex sap fell into oblivion. The term “chicle” (chewing gum) originated from the Maya word “sicte”. The fruit has been used for many purposes in traditional medicine, manufacture of adhesive paints, water resistant varnishes and even for insulation in electric conduction cables [76,77].

Extensive deforestation with the resulting disappearance of many species is not only a loss in biological and economic but also in cultural terms. An important segment of the cultural heritage of indigenous population groups is thus disappearing as is certainly the case throughout the Mayan region. Though not facing extinction per se, as some of them are successfully being cultivated in other parts of the globe [[18], [25], [29], [32], [41], [43]], Mayan NUS have gradually disappeared from their original habitats, including the traditional Mayan backyards to the detriment of the local communities’ diets and to the loss of an integral component of their cultural heritage.

Rescue of some of these NUS (e.g. *Manilkara zapota*) could rely on potential synergies with other sectors, e.g. forestry and environmental sectors. One venue could resort to rewilding ecosystems, which have been subject to destructive human activities [78] by introducing some of the more climate resilient NUS [79]. Efforts have taken place in other parts of Mexico to re-wild various areas which have been subject to intense desertification. What began as a dire need for water in shrinking indigenous communities has now evolved into a revitalization of livelihoods with forest-related, non-timber products bringing new opportunities for income [80]. In a study conducted on 860 native flowering plant species for their commercial potential in an area of 640 ha of a large tropical forest in Los Tuxtlas reserve in the State of Veracruz, Mexico, 22.7% of these were found to produce edible fruits, leaves or flowers [80]. The agricultural knowledge and practice in the pre-European colonization of the Americas is a treasure that has been insufficiently exploited. With the increasing destruction of habitats in the Americas and elsewhere, the preservation of the wide diversity of fruits is a challenge to be undertaken and a must throughout the world, especially in LMICs.

### 4.2 Lessons from other regions of the world

While NUS have attracted relatively little attention in Mexico and the Americas, which is one of the limitations of the current review, they have attracted considerably more attention in other regions of the world. There is a large body of research conducted in different African countries on the domestication of indigenous fruits trees, including their commercialization [81]. There are many lessons to be learnt from these experiences when it comes to applying these to neglected indigenous fruit trees and their products in the Americas and beyond.

Most of these neglected plants have a comparative advantage over the exotic ones. For instance, native species are well adapted to the local conditions and will require less pesticides and fungicides than their exotic counterparts. They evolved to withstand local stress conditions such as drought, herbivory and diseases. These attributes are what make these native plant species particularly attractive as they could contribute to sustainable and more cost-effective production systems [82] at a time when humanity is facing the threats of climate change and desertification [83].

### 4.3 Lessons from the health sector

Many determinants of neglected issues reach well beyond the scope of any one sector alone [1,84]. Taking a non-neglected health topic, the recent COVID-19 pandemic as an example, it soon became clear that any possible attempt to tackle the problem could not possibly rely on the health sector alone, though the pharmaceutical industry played a major role in the development of vaccines, anti-viral drugs and diagnostics. Indeed, the animal health sector, the entire UN system, the economic sector [85] (Ministry of Finance, development banks, etc.), the education and communication sectors, private and public, would all have to pitch in. Neglected issues are no different although their impact might not seem as dramatic as is the case with COVID-19. Saving NUS and their products would make economic, health, cultural and environmental sense; yet, is still neglected.

Issues which are not directly perceived as being profitable by the private sector, e.g. drugs for NTDs [86] coupled with decision makers’ failure to address neglected issues are among the most important underlying factors contributing to the ongoing environmental and health crisis in LMICs [1,84,85]. However successful partnerships involving the public sector, academia, the pharmaceutical industry, WHO and non-governmental organisations have managed to control and in some cases even eliminate NTDs as a public health problem in endemic countries (e.g. lymphatic filariasis, onchocerciasis and trachoma) [87] in some cases by harnessing the collaborative and holistic potential of One Health to reduce their impact [88,89].

### 4.4 Role of civil society

Involvement of civil society is crucial to the success of many programmes, especially those centered around neglected issues. Civil society is in a unique position to offer much needed support for multisectoral collaborations at the ground level. Community-based initiatives represent not only an example of an important botanical and cultural legacy rescue effort, but also provide an example of a successful bottom-up approach. Fortunately, we are already witnessing other groups and local authorities following suit and replicating the project with expertise and seedlings provided by several of its members.

The arboretum project is now looking to expand beyond its urban public park setting to urban gardens and rural communities. Given their exotic nature in terms of flavour, colour, aroma, taste and texture, one should not neglect the important economic potential of the native tree species as a source of pulp and extracts in the production of ice creams both for national consumptions as well as for export [12].

### 4.5 Connection between NUS and health

Indigenous plants are regarded as a huge source of pharmacologically active compounds (Table 1), especially critical during and in the aftermath of the COVID-19 pandemic, while we are now barely realizing the existence of yet another health crisis, that of anti-microbial resistance (AMR) [90]. Antibiotic and anti-fungal agents have been successfully isolated from plants (Table 1). One of the potential pathways towards addressing AMR might lie in finding anti-viral agents in plants [91].

There are relatively few studies on the bioactive compounds of the different parts of native tree species. It appears that their anti-microbial activity relies on the presence of various products such as phenols, phenolic acid, quinones, saponins, flavonoids, tannins, coumarin, terpenoids and alkaloids present in different parts of the plant (Table 1). For example, phenols, alkaloids and terpenoids scavenge free radicals, which have been related to the development and complication of diabetes mellitus [37,92]. Clearly, there is room for improving our understanding of the mechanisms of action of phytochemicals contained in these fruits.

Many of these NUS contain anti-oxidant compounds, which have attracted the attention of the pharmaceutical industry due to their potential effect on aging and their apparent involvement in the prevention of numerous human diseases, including cancer, atherosclerosis, Alzheimer's disease, inflammation, asthma and rheumatoid arthritis [33,21]. Their uses in traditional medicine do cover a surprisingly broad spectrum of perceived benefits (Fig. 1A and Table 1), which depend very much on the geographical location. While some uses tend to repeat themselves across geographical areas, others differ quite a bit. Reasons for this could well depend on whether a fruit is indigenous or imported. Indigenous fruits have had more time to permeate local customs and believes regarding their medicinal properties.

A critical example of the importance and potentials of medicinal plants is the discovery of artemisinin-based combination therapies (ACTs) in the fight against malaria and the timely interventions as the world was witnessing a global crisis of drug resistance to existing anti-malarial drugs. Its recent history goes back to the 1970s [89] with the discovery of the anti-malarial properties of the sweet wormwood leaves (*Artemisia annua*) from the People's Republic of China [93]. At present, ACTs are the drug of choice for malaria treatment in many tropical countries. Some of the largest pharmaceutical companies got involved in what has since become an essential tool in medicine and a highly profitable and essential global enterprise, the development of new drugs from plants. This might now allow us to tackle the emerging challenge of AMR, including anti-fungal resistance [94].

Further studies are needed to enhance our understanding on the mechanisms of action of most of these trees’ bio products. This will require that the private and academic sectors devote more efforts towards the development of much needed new drugs.

There are countless barriers within the commercialization chain of NUS and behind the adoption of NUS to increase the food diversity among the most vulnerable population groups [95]. Nevertheless, we have high hopes that the arboretum project presented here and the open-access book [12], which marks an important milestone of the project, will help disseminate the plight of the neglected native fruit trees and inspire governments, agricultural, environmental and health sectors, and civil society not to forget to rescue this important natural resource to the benefit of local, impoverished communities in LMICs, particularly ethnic minority groups and other vulnerable population groups.

Fortunately, we are beginning to see some breakthroughs in addressing some of the “forgotten” issues in a more holistic and integrated manner. There are no doubts regarding the link between animals, humans and plants and their environment and the countless problems – and opportunities – which arise from these interactions. No one sector alone is in a position to tackle and galvanize most of these. Several sectors must join efforts, synergize rather than antagonize or compete by duplicating actions. Unfortunately, the concept of One Health is still discussed in the context of animal and human health and has rarely ventured beyond that scope in a meaningful way; hence the relevance of this paper. Plant health has received less attention. Nevertheless, interesting discussions have taken place within One Health to strengthen capacity of regulatory bodies by increasing their recognition of the interconnections between people, animals, plants and their shared environment. This is expected to improve the decision-making processes as well as render net benefits from plant protection through food security gains [96]. New research is needed for example on the use of native fruit and vegetable co-products as feed ingredients in farm animal nutrition, particularly relevant in LMICs and on its pharmacological potentials both in human and animal health. Further studies are also needed on its potential climate resilience and natural resistance to pests. One Health could well provide that platform [97,98] to justify and embark in such studies.

## 5 Conclusion

NUS are not only a topic in conservation of a valuable biological resource and thus an element to be considered within the broad scope of environmental health. They could and should follow the same rationale of including pandemic-prone diseases and NTDs, most of which are zoonotic, under the umbrella of One Health. NUS and NTDs share some of the same determinants. Humanity is in dire need for new antibiotics, anti-viral, anti-fungal and anti-parasitic agents in both human and animal health. Yet, there is a lack of perception of profit around NUS as a source of pharmacological agents by the private sector. Our intention with the current paper is to galvanize interest by various sectors at local, national and international levels towards the conservation of NUS. Our case study from Yucatan has thus far proven feasible and successful beyond the involvement of civil society at the community level. Among the project's key selling points was not only the rescue of an important component of Yucatan's cultural heritage but its nutritional value as well as its potential medicinal properties.

The arboretum project has since attracted the attention of various local government sectors (e.g. Major's Office, Department of Sustainable Development, Department of Economic Development and Tourism, Department of Community Involvement, Department of Parks and Public Gardens and Department of Culture). Of note, the Department of Culture acknowledged the group's efforts with an award in 2022 (“Premio Cultura Ciudadana 2022, Categoria: Convivencia con el Entorno Ambiental”). Furthermore, the arboretum was nominated to become a member of Merida's museum network as of June 2023. The local authorities are now actively advocating for the establishment of similar projects in other parts of the city of Merida and disseminating information of the project in government digital platforms. Some of the fruit trees are now included in the municipality's inventory of tree nurseries to be donated to the population, while the local academic sector is inviting the group to share its experiences. Polices should gradually follow as the involvement of the authorities and the civil society increase. The prospects are promising. This multi-sectorial involvement opens the door to the involvement of the health sector under the One Health platform. The opportunity to do so lies now within the realm of the academic and public sector and possibly of the various UN agencies promoting One Health, such as FAO, WHO, World Organisation for Animal Health, United Nations Environment Programme and United Nations Development Programme.

Our paper is by no means intended as an all-inclusive prescription on how NUS are to be articulated within the One Health umbrella or other strategies. It is merely meant as a thought-provoking piece on why it is important to take advantage of the One Health platform. Our article should stimulate more interest in the subject through small-scale projects the likes of the one presented in this manuscript by members of the civil society in Yucatan and the authors of this paper and more action on the side of academia and the pharmaceutical sector on the wide reaching potential of NUS.

## Funding

This research did not receive any specific grant from funding agencies in the public, commercial or not-for-profit sectors.

## CRediT authorship contribution statement

**John P. Ehrenberg:** Writing – review & editing, Writing – original draft, Methodology, Formal analysis, Data curation, Conceptualization. **Afona Chernet:** Writing – review & editing, Supervision, Resources, Methodology, Investigation. **Manuel Luján:** Writing – review & editing, Visualization, Methodology, Investigation. **Jürg Utzinger:** Writing – review & editing, Supervision, Resources, Methodology, Investigation, Formal analysis.

## Declaration of competing interest

The authors declare that they have no known competing financial interests or personal relationships that could have appeared to influence the work reported in this article.
